# Primary malignant non-Hodgkin’s lymphoma of the breast: A case report

**DOI:** 10.3892/ol.2014.2612

**Published:** 2014-10-10

**Authors:** YUN-FEI ZHAO, FENG JIAO, HAI-QIAO LIANG, QI-CHI LUO, LIN-WEI ZHAO

**Affiliations:** 1 Department of Pathology, Suining Central Hospital, Suining, Sichuan 629000, P.R. China; 2 Department of Oncology, Shanghai Jiao Tong University Affiliated First People’s Hospital, Shanghai 201620, P.R. China; 3 Department of Radiology, Suining Central Hospital, Suining, Sichuan 629000, P.R. China

**Keywords:** malignant lymphoma, breast lymphoma, immunohistochemistry

## Abstract

Primary malignant lymphoma of the breast (PLB) is a rare disease. Treatment options include surgical resection, systemic chemotherapy, radiation and immunotherapy. At present, the optimum treatment combination remains controversial. The present study reports the case of a 39-year-old female with a six month history of a painless mass in the left breast. The mass was excised following medical examination. A diagnosis of diffuse large B-cell lymphoma was determined as a result of histological and immunohistochemical profile analysis. Further examinations excluded metastatic disease. Thus, finally, PLB (diffuse large B-cell lymphoma type) was diagnosed. The patient was treated with adjuvant systemic chemotherapy and consolidated radiation and a positive response was observed. During the 10 months of follow-up, no evidence of disease recurrence was identified. At present, the patient is scheduled for regular follow-up appointments. As the prevalence of PLB is increasing, the details of this rare case may aid clinicians treating similar patients, and highlight the importance of this disease.

## Introduction

Primary malignant lymphoma of the breast (PLB) is a rare disease, which accounts for only 0.4–0.5% of all breast malignancies, 0.38–0.7% of all non-Hodgkin’s lymphomas (NHLs) and 1.7–2.2% of extranodal NHLs in the caucasian population (1–4). A painless mass is the most common presentation, which occurs in ~61% of cases (5). Other symptoms include palpable lymph nodes, local pain and local inflamation (5). The majority of cases of PLB are diagnosed by biopsy or postoperative pathological observations. The specific criteria for the diagnosis of PLB includes, the breast as the tumor site, a history of previous lymphoma and no evidence of widespread disease at diagnosis, lymphoma has been demonstrated to exhibit a close association with breast tissue in pathological specimens, and ipsilateral lymph node involvement (6). Current treatments for PLB include radiotherapy and/or chemotherapy (5). The use of combined therapy is considered to be the most effective for PLB patients, even at the early stages of the disease (1). The overall prognosis of patient with PLB is relatively good, with an overall five-year survival rate of 50–82% (7–9). The present study reports the rare case of 39-year-old female with PLB. Due to the rarity of the disease, the relevant literature was also reviewed. Written informed consent was obtained from the patient.

## Case report

**Figure 1:**
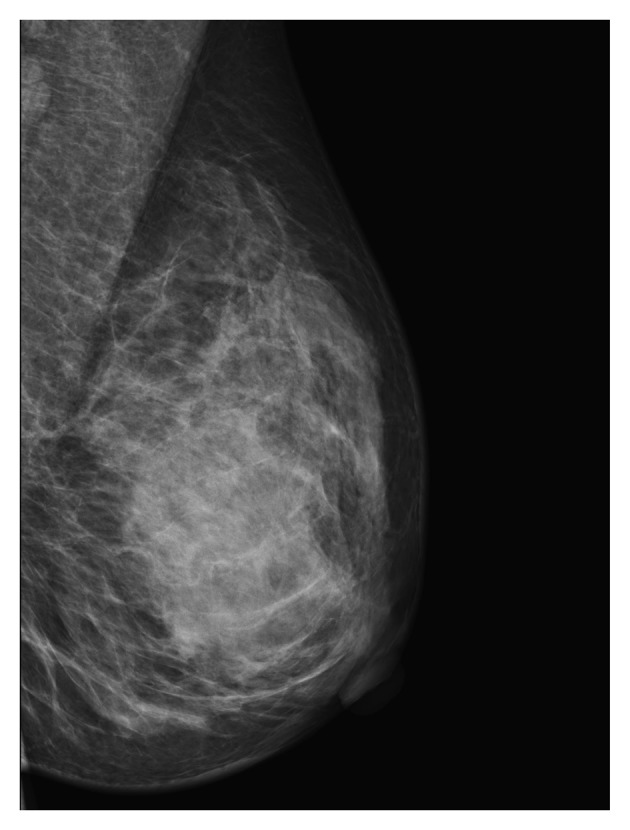
Digital radiography examination revealed a 5.0×4.0×4.0 cm mass with an increased density shadow in the left breast.

**Figure 2:**
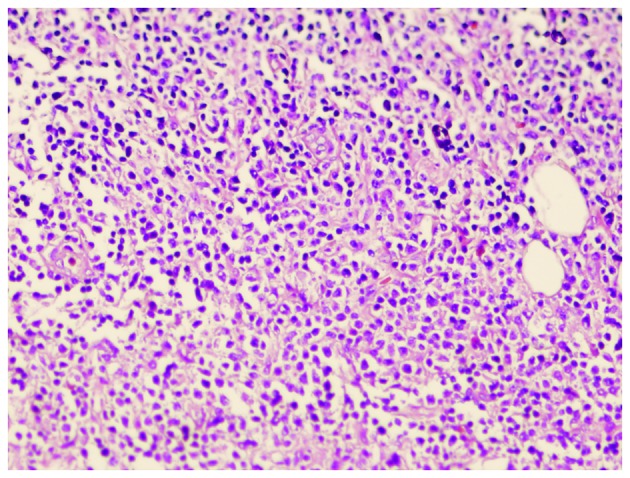
Histological examination results confirmed a malignant lymphoma. The diffuse infiltration of numerous of neoplastic lymphocytes was identified (hematoxylin-eosin stain; magnification, ×100).

**Figure 3:**
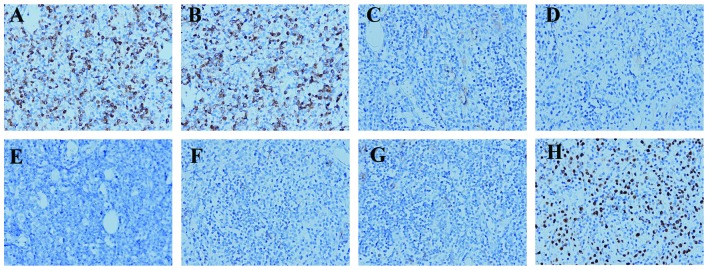
Immunohistochemical profile analysis revealed a large B cell lymphoma (magnification, ×200), which was positive for (A) CD20 and (B) CD79a, negative for (C) CD3, (D) myeloperoxidase, (E) terminal deoxynucleotidyl transferase, (F) CD99 and (G) CD138, and (H) positive for Ki-67. CD, cluster of differentiation.

In April 2013, a 39-year-old female presented to the Department of Surgery, Suining Central Hospital (Suining, China) with a six month history of a painless mass in the left breast. The mass had rapidly increased in size over six months. No history of other diseases was identified. On physical examination, a nontender, demarcated firm 5.0×5.0 cm elastic mass with an irregular surface, was palpable in the upper inner quadrant of the left breast. The right breast was normal. Enlargement of the axillary lymph nodes was not identified. On ultrasonography, the mass was observed to be solid, almost entirely hyperechoic, and exhibited a circumscribed margin in the palpable area. Digital radiographic examination revealed a mass of 5.0×4.0×4.0 cm with an increased density shadow (Fig. 1). The radiological results were assessed prospectively according to the American College of Radiology Breast Imaging-Reporting and Data System (10,11) as category 3 or 4 (suspicion for malignancy). An ultrasound guided core needle biopsy of the left breast was performed. The histological results revealed the infiltration of a large number of lymphocytes into the breast lobular and duct and lymphocyte hyperplastic lesions were suspected. Thus, mass excision was performed and a definitive diagnosis was established. Grossly, the 5.0×5.0×4.0 cm tumor was gray-white and poorly circumscribed. Microscopically, the tumor cells demonstrated invasive growth and a tendency to surround and invade the wall and lumina of the epithelial structures, resulting in a lymphoepithelial lesion. In addition, the mammary gland structure was destroyed. Numerous neoplastic lymphocytes revealed a diffuse growth pattern (Fig. 2). The examination results revealed malignant lymphoma and thus, further immunophenotype analysis was required to determine the type of lymphoma. The immunohistochemical profile was positive for cluster of differentiation (CD)20 and CD79a and negative for CD3, myeloperoxidase, terminal deoxynucleotidyl transferase, CD99 and CD138 (Fig. 3). In addition, the Ki67 positive rate was 60% (Fig. 3). The results confirmed the diagnosis of a diffuse large B-cell lymphoma. Additionally, the patient underwent further examination to exclude metastatic disease using positron emission tomography/computed tomography, which revealed no evidence of further disease. Thus, primary NHL of the breast (diffuse large B-cell lymphoma type) was diagnosed. The patient was treated with six cycles of combination chemotherapy [intravenous cyclophosphamide (750 mg/m^2^, day 1), intravenous doxorubicin (50 mg/m^2^, day 1), intravenous vincristine (1.4 mg/m^2^, day 1 and 8) and prednisone (80 mg, daus 1–5)] for six months. Following three cycles of chemotherapy, radiation was adminstered to the local site (40 Gy), in combination with chemotherapy for an additional three cycles. The patient exhibited a positive response with no evidence of disease. During the follow-up period of 10 months, no symptoms or signs of disease recurrence were observed. At present, the patient is receiving regular follow-up.

## Discussion

PLB remains a rare disease, however, the occurrence is increasing due to improvements to diagnostic techniques and increasing awareness of the disease. The criteria for PLB diagnosis have been defined by Wiseman and Liao (12), and include an adequate pathological specimen, presence of mammary tissue and lymphomatous infiltrate in close association and exclusion of previous extramammarian lymphoma or systemic lymphoma. The presence of ipsilateral axillary lymph node involvement has also been considered acceptable criteria (13). Histologically, PLB may be grouped as a large cell B-cell lymphoma, monocytoid B-cell lymphoma, and undifferentiated, some of which may be T-cell (14). The histological examination and immunohistochemical profile may aid with differentiating between breast diseases, including medullary carcinoma and invasive lobular carcinoma. According to the aforementioned criteria, in the present study, the patient was diagnosed with PLB. The immunohistochemical analysis revealed large B-cell lymphoma.

To date, no standard treatment for PLB has been identified. Mastectomy is not indicated and wide local excision is not required as these tumors are highly sensitive to radiotherapy and systemic chemotherapy (15). In a previous study, combined therapy using chemotherapy and radiotherapy, was been found to be the most successful treatment (16). For small localized tumors, adequate surgical resection may be effective, followed by chemotherapy or radiotherapy (12). With regards to prognosis, it appears that patients with PBL exhibit a better prognosis than patients with breast cancer or/and other extranodal lymphomas (17). Furthermore, previous studies have indicated that the size of PBL, bilaterality and axillary lymph node involvement did not affect prognosis, however, the histological type appeared to be the most significant prognostic factor, with low-grade PBL exhibiting the best prognosis and high-grade exhibiting the poorest prognosis (14,18).

However, the majority of studies have been descriptive studies and thus, detailed studies exploring the molecular mechanisms of PLB are urgently required. A number of hypotheses have been proposed to explain the pathogenesis of PLB. Hormonal stimulation may be an important factor as PLB was observed frequently in women during pregnancy or postpartum (1). In male patients, the administration of steroid hormones was associated with the manifestation of PLB. In addition, certain studies have revealed a clear association between PBL and mucosa-associated lymphoid tissue (19). The detailed etiology and mechanism of PLB origination and progression requires further clarification.

In conclusion, this study reports a rare case of a female with PLB presenting as a painless mass in the left breast. As the clinical symptoms of PLB are diverse and nonspecific, misdiagnosis as other breast diseases may occur. Overall, PLB is rare, however, the prevalence is increasing. Therefore, this disease requires attention. The early diagnosis and timely treatment are important factors when treating PLB. Furthermore, additional studies focusing on the etiology and mechanism of PLB are required.
